# Science and policy in substance abuse

**DOI:** 10.1186/1747-597X-1-1

**Published:** 2006-01-12

**Authors:** Stephan Arndt

**Affiliations:** 1 Professor, Department of Psychiatry, Carver College of Medicine, University of Iowa, Iowa City, IA 52242, USA; 2 Professor, Department of Biostatistics, College of Public Health, University of Iowa, Iowa City, IA 52242, USA; 3 Director, Iowa Consortium for Substance Abuse Research and Evaluation, Iowa City, IA 52242, USA

## Abstract

*Substance Abuse Treatment, Prevention, and Policy *(*SATPP*) is an open access, peer-reviewed international journal of original research and scholarship that focuses on policy issues in the treatment and prevention of substance use disorders. Separate and often disparate public systems deal with substance use problems as well as provide treatment and prevention. This journal will provide an environment for the exchange of ideas, new research, consensus papers, and critical reviews that bridge fields that share a common goal of reducing the problems caused by drugs and alcohol. The agenda is simple; a new forum for integrating thoughts, issues, and developments.

## 

Separate and often disparate public systems deal with substance abuse as well as provide substance abuse treatment and prevention. These include: legislation pertaining to substance abuse; correctional supervision of substance abusers; medical treatment and screening; mental health services; research and evaluation of substance abuse programs; interdiction efforts, and so on. These varied systems share a common goal – to reduce the burden of substance abuse on the abuser, their family, and society. Unfortunately, these systems often operate in isolation of one another. This journal will provide an environment for the exchange of ideas, new research, consensus papers, and critical reviews that bridge fields that share a common goal of reducing the problems caused by substance abuse.

## What kinds of papers interest us?

We are interested in articles on research and evaluation of issues pertaining to treatment, recovery, and prevention, but with an emphasis on items such as: evidence-based practices; stigma reduction; how public policy and law making have implications for treatment and recovery; removing barriers to treatment and recovery; how decision makers can implement new treatment practices; and the epidemiological trends in substance abuse in special populations (e.g., corrections offenders, women, elders). We will consider original data driven articles, consensus topics, literature reviews – all with a policy flavor. These papers may come from, but are not limited to, psychology, psychiatry, sociology, medicine, social work, law, forensics, corrections, education, health policy, and public health.

There are many potential papers that go unpublished in the scholarly literature that can find a home here. For example, the Robert Woods Johnson Foundation "Substance Abuse Policy Research Program" funds a very large number of projects that would yield perfectly relevant and potentially publishable findings .

Governmental agencies also receive large numbers of reports such as program evaluations, some brand of cost-benefit analysis, and position papers regarding substance abuse, a portion of which are relevant and excellent. For example, independent units submit many data driven reports a year, usually to state agencies, most of which have policy relevance. My guess is that many agencies receive an abundance of policy-related reports. The task here will be to solicit groups to submit high quality publications based on these reports. Finally, we purposefully included prevention into our mix since this is a highly untapped area. Broadening the scope a bit, most papers on substance abuse, with the exception of wet lab-work, have policy relevance because of the current social marginalizing disposition of substance use.

Our interest in policy is international and definitely not confined to U.S. policies. It is very true that the current public policy on substance use and abuse in the US is different from many parts of the world. However, having region specific papers, U.S. and others, may be of general interest. An international discussion will enrich the journal's content. Furthermore, policies in one country affect policies elsewhere.

## What do we mean by policy?

We envision the term "policy" to be broad and not just public, governmental, or legal policy. Policies include issues with clinical practice, the definitions of abuse, cultural perspectives (e.g., regarding stigma, language, parity), or other issues. For example, a current policy in the practice of treatment may be for individuals to undergo a detoxification (detox) period of about three days. This practice likely comes from alcohol addiction and allows treatment staff to closely monitor the person during the time when delirium tremens or seizure might occur. The three-day time period may not apply to other substances of abuse (e.g., marijuana, stimulants) but in practice the detox period remains the same. It would be interesting for someone to review the purpose of the detox period or even review how detox is done in other regions or situations. Another policy example regards the separation of substance abuse treatment from mental health treatment. That separation is widely prevalent in the US and likely occurs elsewhere, too. More generally, governmental and healthcare system policies affect the delivery, access, and process of both treatment and prevention.

Policies also affect our definitions of abuse (DSM IV; the American Psychiatric Association's Diagnostic and Statistical Manual of Mental Disorders) or harmful/hazardous use (ICD 10; the World Health Organization's International Classification of Diseases), since policies can create the negative consequences. The consequences can vary from mild social disapproval, loss of employment, or incarceration and vilification. Few other medical diagnoses appearing in DSM or ICD have this distinction.

So, we welcome you to *Substance Abuse Treatment, Prevention, and Policy*. The journal will strive for impartiality and fast turn around time. An open access mechanism particularly fits the journal's needs by broadening our readership throughout the world as well as speeding the dissemination of manuscripts. We are proud of our editorial board, which represent a very wide group of individuals from numerous disciplines and regions. The agenda is simple, a new forum for integrating thoughts, issues, and developments.

